# Invasive *Salmonella* *enterica* Serotype Typhimurium Infections, Democratic Republic of the Congo, 2007–2011

**DOI:** 10.3201/eid2004.131488

**Published:** 2014-04

**Authors:** Benedikt Ley, Simon Le Hello, Octavie Lunguya, Veerle Lejon, Jean-Jacques Muyembe, François-Xavier Weill, Jan Jacobs

**Affiliations:** Institute of Tropical Medicine, Antwerp, Belgium (B. Ley, J. Jacobs);; Institut Pasteur, Paris, France (S. Le Hello, F.-X. Weill);; Institut National de Recherche Biomédicale, Kinshasa, Democratic Republic of the Congo (O. Lunguya, J.-J. Muyembe);; Institut de Recherche pour le Développement, Montpellier, France (V. Lejon)

**Keywords:** *Salmonella* Typhimurium, ST313, CRISPOL, MLST, Central Africa, bacteremia, bacteria, Democratic Republic of the Congo

## Abstract

Infection with *Salmonella enterica* serotype Typhimurium sequence type (ST) 313 is associated with high rates of drug resistance, bloodstream infections, and death. To determine whether ST313 is dominant in the Democratic Republic of the Congo, we studied 180 isolates collected during 2007–2011; 96% belonged to CRISPOL type CT28, which is associated with ST313.

*Salmonella enterica* serotype Typhimurium multilocus sequence type (ST) 313 has been reported as an emerging cause of invasive salmonellosis in sub-Saharan Africa (*1*). ST313 is almost exclusively from sub-Saharan Africa, is characterized by a degraded genome capacity similar to that of *S. enterica* Typhi, has high rates of antimicrobial drug resistance, and is associated with bloodstream infections and mortality rates >25% (*2*). Whole-genome sequence analysis of 129 ST313 strains, isolated during 1988–2010 from 7 countries of sub-Saharan Africa, identified 2 dominant genetic lineages, I and II. These lineages emerged ≈52 and ≈32 years ago, respectively, possibly coevolving with the spread of HIV (*3*). Although lineage I has not been observed since the mid-2000s, lineage II has been observed with increasing frequency. However, data from Central Africa, particularly the Democratic Republic of the Congo (DRC) are scarce, and information is limited to 10 genomes from strains isolated >20 years ago (*3*). To determine whether ST313 is the dominant ST among invasive *S. enterica* Typhimurium in the DRC, we studied 180 isolates collected during 2007–2011.

## The Study

**Figure 1:**
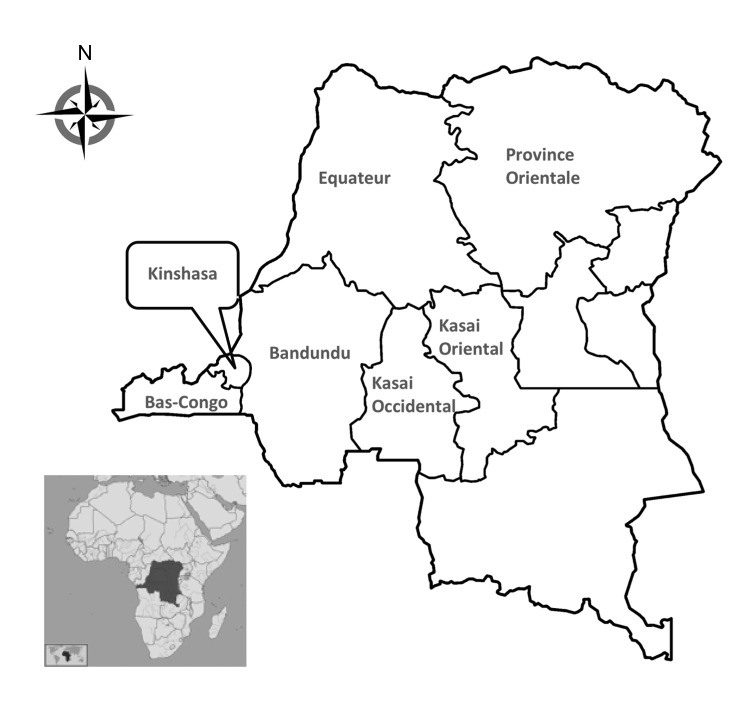
Provinces with sample collection sites in the Democratic Republic of the Congo, 2007–2011(*4*).

We earlier described a series of invasive non-Typhi *S. enterica* isolates from blood cultures collected in 7 of the 11 provinces in the DRC during 2007–2011 (Figure 1) (*4*). In that study, a health care facility–based survey was administered to persons who met the eligibility criteria of suspected bacteremia at time of presentation and patient age >2 months. Blood culture vials were shipped to Kinshasa and processed according to standard identification procedures (*4*). A total of 233 non-Typhi *S. enterica* isolates were recovered, 184 (79%) of which belonged to serotype Typhimurium (*4*). The serotypes for all *S. enterica* Typhimurium isolates were determined locally and later confirmed at the Institute of Tropical Medicine, Antwerp, Belgium. Most (180/184, 98.7%) *S. enterica* Typhimurium isolates were subsequently shipped to the Pasteur Institute, Paris, France, for further analysis.

The population structure of these 180 *S. enterica* Typhimurium isolates was assessed by CRISPOL typing. CRISPOL is a recently developed high-throughput assay based on clustered regularly interspaced short palindromic repeat (CRISPR) polymorphisms (*5*). This bead-based hybridization assay is designed to detect the presence or absence of 72 short variable DNA sequences (spacers) from both CRISPR loci of *S. enterica* Typhimurium. Initially, 245 different CRISPOL types (CTs) were identified in a 2012 study that included 2,200 isolates (*5*); just before we conducted the study reported here, the CRISPOL *Salmonella *Typhimurium database of the Pasteur Institute contained >7,000 strains comprising >750 different CTs.

**Figure 2:**
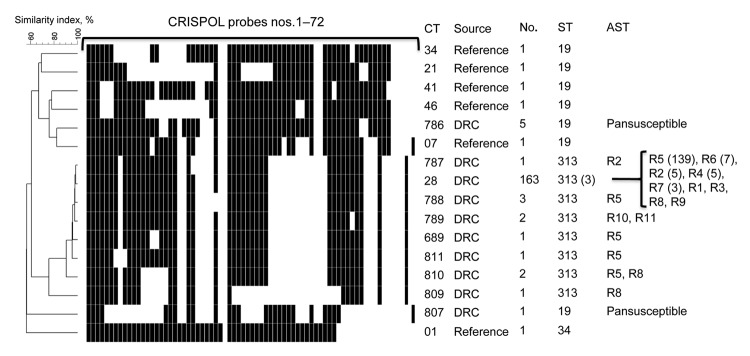
Representative CRISPOL profiles of *Salmonella enterica* serotype Typhimurium isolates studied. CRISPOL is a recently developed high-throughput assay based on clustered regularly interspaced short palindromic repeats (CRISPR) polymorphisms. Black squares indicate presence of the CRISPR spacer, detected by the corresponding probe; white squares indicates absence of the spacer. The dendrogram was generated by using BioNumerics version 6.6 software (Applied Maths, Sint-Martens-Latem, Belgium) as described (*5*). The CRISPOL types (CTs) detected among the 180 isolates from the Democratic Republic of Congo (DRC) are labeled as DRC in the Source column. Six common CTs of the Pasteur Institute CRISPOL database (labeled as reference) are also shown. These CTs are from strains of serotype Typhimurium 02–1800 (CT34, DT120), 02–5270 (CT21, DT104), LT2 (CT41, DT4), 02–2561 (CT46, DT12), 02–1749 (CT7, DT14) or its monophasic variant of antigenic formula 1,4,[5],12:i:-, 07–1777 (CT1, DT193). For each distinct CT, the numbers of corresponding isolates, their sequence types (STs), and their antimicrobial drug susceptibility testing (AST) data are indicated. For the ST and AST columns, the numbers in parentheses refer to the number (>2) of tested isolates with such result. AST data are shown only for DRC isolates. The resistance types were as follows: R1, ASKTNGSulTmpC; R2, ASKTNGSulTmpCTe; R3, AC; R4, ASSulTmp; R5, ASSulTmpC; R6, ASSulTmpCNal; R7, ASSulTmpCTe; R8, ASulTmpC; R9, SSulTmpC; R10, ACroCazSKTNGSulTmpCTeAzi; and R11, ACroSKTNGSulTmpCTeNaAzi. Abbreviations used in the descriptions of resistance types are as follows: A, amoxicillin; Cro, ceftriaxone; Caz; ceftazidime; S, streptomycin; K, kanamycin; T, tobramycin; N, netilmicin; G, gentamicin; Sul, sulfamethoxazole; Tmp, trimethoprim; C, chloramphenicol; Te, tetracycline; Nal, nalidixic acid; Azi, azithromycin.

A total of 174 (96.7%) *S. enterica* Typhimurium isolates from the DRC belonged to the CT28 group, of which 163 (90.5%) were CT28. A total of 11 (6.1%) isolates belonged to 7 other CTs that were single-spacer variants (loss of a single spacer), single-event variants (loss of >2 contiguous spacers), or double-event variants of CT28 (Figure 2). Six (3.3%) isolates belonged to 2 CTs not related to CT28. CT28 had been associated with ST313 in a multidrug-resistant DT56 *S. enterica* Typhimurium isolate from Senegal and in the D23580 ST313 lineage II genome (*5*). In contrast, the analysis of raw pyrosequence data for genome A130 (*3*), representative of ST313 lineage I, corresponded to CT698, distinct from the CT28 group.

To confirm the association of ST313 to the CT28 group, we performed multilocus sequence typing (MLST) (*6*) on 12 isolates. A total of 3 isolates belonged to CT28, and 1 isolate of each single-spacer, single-event, and double-event variant was tested, resulting in 10 isolates from the CT28 group. We also performed MLST on 1 isolate of each of the 2 non–CT28 group isolates. All 10 CT28 group isolates tested were ST313; both non–CT28 group isolates tested were ST19.

Antimicrobial drug susceptibility has been studied with a limited panel of 7 drugs (*4*). We performed additional susceptibility testing by disk diffusion with a panel of 32 antimicrobial agents (Bio-Rad, Marnes-La-Coquette, France) (*7*). Extended-spectrum β-lactamase (ESBL) phenotype was assessed by using the double-disk synergy method (*8*). For all ESBL-producing isolates, MICs of ceftriaxone, ceftazidime, azithromycin, and imipenem were determined by the Etest macromethod (bioMérieux, Marcy L'Etoile, France). Results were interpreted according to break points defined by the Antibiogram Committee of the French Society for Microbiology (www.sfm-microbiologie.org/). Susceptible strains were defined as having a ceftriaxone MIC <1 mg/L, ceftazidime MIC <4 mg/L, azithromycin MIC <16 mg/L, and imipenem MIC <2 mg/L. Resistance was defined as having a ceftriaxone MIC >2 mg/L, ceftazidime MIC >4 mg/L, azithromycin MIC >16 mg/L, and imipenem MIC >8 mg/L. The presence of macrolide resistance genes was assessed by PCR and sequencing as described elsewhere (*7*). Of the 174 CT28 group isolates, 167 (96%) were resistant to ampicillin, chloramphenicol, and trimethoprim/sulfamethoxazole in combination with other drugs (Figure 2); the remaining isolates were resistant to 1 or 2 of these drugs. Two isolates were resistant to extended-spectrum cephalosporins (ceftriaxone MIC 6–32 mg/L, ceftazidime MIC 4–32 mg/L); both contain the ESBL *bla*_SHV-2a_ gene (*4*). We report that both isolates contain the *mph*(A) gene encoding a macrolide 2′-phosphotransferase that inactivates macrolides (azithromycin MIC 96–128 mg/L). All 6 non-CT28 group isolates were susceptible to all drugs tested (Figure 2).

## Conclusion

Our data are based on the analysis of *S. enterica* Typhimurium isolates recovered from >9,600 blood cultures collected during a 4-year period from distinct parts of the DRC. We found that >96% of the *S. enterica* Typhimurium isolates belonged to the CT28 group. Because of the strong association between CT28 group and ST313, our findings suggest high rates of ST313 among invasive salmonellosis in the DRC.

Of the 10 genomes from the DRC isolated during 1988–1992 (*3*), genetic lineages I and II were identified at approximately equal rates. Of the more recent isolates (2007–2011) described here, all ST313 isolates belonged to the CT28 group, associated with lineage II. A notable feature of lineage II is chloramphenicol resistance resulting from a *cat* gene within a specific Tn21-like element, carried by the virulence-associated plasmid pSLT (*3*). In the set described herein, we observed chloramphenicol resistance in >97% of all isolates belonging to the CT28 group. The almost complete replacement of lineage I isolates by lineage II isolates from Kenya and Malawi has also been reported (*1*,*9*).

Our data are based on invasive *S. enterica* Typhimurium isolates collected in a nonsystematic health care facility–based approach and do not include noninvasive strains of *S. enterica* Typhimurium. Wain et al. (*10*) recently cited unpublished data showing that ST313 *S. enterica* Typhimurium might be a common cause of gastroenteritis among immune-competent patients. A human reservoir for multidrug-resistant *S. enterica* Typhimurium and Enteritidis in Kenya has been suggested because of the presence of similar strains in asymptomatic siblings and parents of index case-patients (carriage prevalence 6.9%) (*11*). Whole-genome sequencing of ST313 strains has shown genome degradation, including pseudogene formation and chromosomal deletions as have been observed for human-restricted *S. enterica* Typhi (*12*,*13*), suggesting that ST313 might be undergoing an evolution toward niche specialization or, more likely, human adaptation (*1*).

Our results indicate very high rates of multidrug-resistant *S. enterica* Typhimurium ST313 among invasive non-Typhi *Salmonella* infections in the DRC. Future field studies involving patients with uncomplicated *Salmonella* spp. infections will help determine whether ST313 *S. enterica* Typhimurium in Central Africa is an opportunist or a primary pathogen. Systematic analyses of potential nonhuman and human reservoirs of *S. enterica* Typhimurium might provide a better understanding of the transmission dynamics of this emerging pathogen.
